# Determining Risk for Severe Leptospirosis by Molecular Analysis of Environmental Surface Waters for Pathogenic *Leptospira*

**DOI:** 10.1371/journal.pmed.0030308

**Published:** 2006-08-22

**Authors:** Christian A Ganoza, Michael A Matthias, Devon Collins-Richards, Kimberly C Brouwer, Calaveras B Cunningham, Eddy R Segura, Robert H Gilman, Eduardo Gotuzzo, Joseph M Vinetz

**Affiliations:** 1 Instituto de Medicina Tropical Alexander von Humboldt, Universidad Peruana Cayetano Heredia, Lima, Peru; 2 Division of Infectious Diseases, Department of Medicine, University of California San Diego School of Medicine, La Jolla, California, United States of America; 3 Division of International Health and Cross-Cultural Medicine, Department of Family and Preventive Medicine, University of California San Diego, La Jolla, California, United States of America; 4 Department of International Health, Johns Hopkins Bloomberg School of Public Health, Baltimore, Maryland, United States of America; Mahidol University, Thailand

## Abstract

**Background:**

Although previous data indicate that the overall incidence of human leptospirosis in the Peruvian Amazon is similar in urban and rural sites, severe leptospirosis has been observed only in the urban context. As a potential explanation for this epidemiological observation, we tested the hypothesis that concentrations of more virulent *Leptospira* would be higher in urban than in rural environmental surface waters.

**Methods and Findings:**

A quantitative real-time PCR assay was used to compare levels of *Leptospira* in urban and rural environmental surface waters in sites in the Peruvian Amazon region of Iquitos. Molecular taxonomic analysis of a 1,200-bp segment of the leptospiral 16S ribosomal RNA gene was used to identify *Leptospira* to the species level. Pathogenic *Leptospira* species were found only in urban slum water sources (Fisher's exact test; *p* = 0.013). The concentration of pathogen-related *Leptospira* was higher in urban than rural water sources (~10^3^ leptospires/ml versus 0.5 × 10^2^ leptospires/ml; F = 8.406, *p* < 0.05). Identical 16S rRNA gene sequences from Leptospira interrogans serovar Icterohaemorrhagiae were found in urban slum market area gutter water and in human isolates, suggesting a specific mode of transmission from rats to humans. In a prospective, population-based study of patients presenting with acute febrile illness, isolation of L. interrogans-related leptospires from humans was significantly associated with urban acquisition (75% of urban isolates); human isolates of other leptospiral species were associated with rural acquisition (78% of rural isolates) (chi-square analysis; *p* < 0.01). This distribution of human leptospiral isolates mirrored the distribution of leptospiral 16S ribosomal gene sequences in urban and rural water sources.

**Conclusions:**

Our findings data support the hypothesis that urban severe leptospirosis in the Peruvian Amazon is associated with higher concentrations of more pathogenic leptospires at sites of exposure and transmission. This combined quantitative and molecular taxonomical risk assessment of environmental surface waters is globally applicable for assessing risk for leptospiral infection and severe disease in leptospirosis-endemic regions.

## Abstract 2

Vinetz and colleagues used a quantitative real time PCR assay combined with molecular taxonomic analysis to quantify *Leptospira* in environmental surface waters in the Peruvian Amazon region of Iquitos.

## Editors' Summary

**Background.**

Humans catch many diseases from animals—so-called zoonotic infections. Often, these occur in limited regions of the world. However, one—leptospirosis—occurs in temperate and tropical climates, and in urban and rural settings, making it the most widespread zoonotic disease. Leptospirosis is caused by *Leptospira,* a large group of closely related spiral-shaped bacteria that live in both domestic animals (for example, cattle) and wild animals (particularly rats). Millions of humans become infected each year with leptospires through close contact with water, food, or soil contaminated with the urine of infected animals—swimming or wading in contaminated water is particularly hazardous. Some infected people have no symptoms; others develop a flu-like disease that clears up within a few days. However, in 5%–10% of infected people, the disease progresses to a second, sometimes fatal phase. This is usually characterized by jaundice, kidney problems, and an enlarged spleen (it's then called Weil disease) but can also involve the lungs (pulmonary leptospirosis). Leptospirosis can be successfully treated with antibiotics if treatment is started soon after infection.

**Why Was This Study Done?**

In a recent study in the Peruvian Amazon, half of the people visiting urban hospitals and rural health posts with acute fever had antibodies in their blood to *Leptospira,* suggesting that they had acute leptospirosis. However, only patients living in urban areas developed pulmonary leptospirosis. In this study, the researchers tested the hypothesis that this pattern arose because more virulent types of *Leptospira* were present at higher levels in urban environmental surface water than in rural water sources.

**What Did the Researchers Do and Find?**

Between June 2003 and March 2004, the researchers isolated strains of *Leptospira* from patients with acute fever who visited a hospital in the town of Iquitos or clinics in nearby villages. Early in 2004, they also collected a large number of different water samples from an urban slum in Iquitos and from a nearby rural community. They measured the concentrations of *Leptospira* in these samples by using a molecular technique called real-time PCR (polymerase chain reaction) to detect and quantify a type of RNA found only in disease-causing *Leptospira*. They also identified which specific *Leptospira* were present in the water samples and the patient samples by sequencing this RNA. The researchers found that leptospires were present in both urban and rural water samples (particularly in samples from gutters and puddles in the urban slum's market area) but that their concentration in the positive water samples from the urban sites was 20 times that in the positive samples from the rural sites. Furthermore, the distribution of different *Leptospira* types isolated from the patients mirrored that of the bacteria in the local environment. So, one particular type of Leptospira interrogans known as icterohaemorrhagiae—the leptospire most commonly associated with severe leptospirosis in the patients—was found more often in the urban water samples than in the rural ones. Finally, the researchers discovered a new group of *Leptospira* in the rural environment. This group may contain one or several new species of *Leptospira* but whether any of them causes human disease is unknown.

**What Do These Findings Mean?**

These results support the researchers' hypothesis that pulmonary leptospirosis in urban areas of the Peruvian Amazon is associated with high environmental levels of specific disease-causing leptospires. The researchers were able to discover this link only by using molecular techniques—this sort of study is impossible with traditional bacteriological techniques because *Leptospira* are hard to grow in the laboratory and cannot be isolated efficiently from environmental water sources. Different types can't be identified using a microscope. The researchers' findings need to be validated in other settings, but they suggest that environmental interventions such as reducing sources of standing water and clearing away garbage in urban areas might reduce the number of cases of severe leptospirosis. The distribution of different *Leptospira* types also suggests that whereas rats may be the main disease reservoir in towns, cattle, pigs, and bats may be more important in rural settings in Peru and presumably elsewhere. Overall, this new information, together with the availability of molecular methods for rapid clinical diagnosis and environmental risk assessment, should aid attempts to control leptospirosis around the world.

**Additional Information.**

Please access these Web sites via the online version of this summary at http://dx.doi.org/10.1371/journal.pmed.0030308.

- US Centers for Disease Control and Prevention, information for patients and professionals on leptospirosis
- The Leptospirosis Information Center, information and advice on human leptospirosis for the public and medical professionals
- MedlinePlus encyclopedia entry on leptospirosis
- NHS Direct Online, patient information on leptospirosis from the UK National Health Service online encyclopedia
- Wikipedia pages on leptospirosis (note: Wikipedia is a free online encyclopedia that anyone can edit)

## Introduction

Leptospirosis is a globally important zoonotic disease caused by spirochetes from the genus *Leptospira*. Annually, tens of millions of human cases occur worldwide, with case fatality rates ranging as high as 20%–25% in some regions [1]. Leptospirosis is transmitted to humans through environmental surface waters contaminated by the urine of domestic and wild mammals chronically colonized with *Leptospira* [2]. Leptospirosis occurs in both industrialized and developing countries [3–5], but is particularly prevalent in tropical countries where environmental and socioeconomic conditions for its transmission are particularly favorable [6–10]. Leptospires can survive in warm, moist soil and in water for weeks to months [11]. In tropical developing countries, poor people typically live either under highly crowded conditions or in rural places. Both types of environments facilitate human exposure to the urine of *Leptospira*-infected mammals, whether from livestock, companion animals, peridomestic rodents, or wild animals. Notably, rats flourish in urban areas of both developing and industrialized countries, providing substantial opportunity for rodent-borne leptospires to infect people [5,7]. Recent outbreaks worldwide among athletes, military personnel, and civilians highlight the risk to travelers for acquiring leptospirosis in tropical environments [12,13]. Furthermore, noted risk factors, including the use of well or stream water, minding livestock, walking barefoot, and the presence of rats and cats at home, have been shown to be associated with being exposed to *Leptospira* [12–15]. Transmission also appears to coincide with warm weather and the occurrence of severe weather and flooding, which washes soil contaminated with animal urine into water sources of potential human use [7,16–20].

We recently demonstrated that in the region of Iquitos, Peru, severe pulmonary leptospirosis was associated with urban acquisition of the pathogen [9]; we observed that approximately half of all cases of acute febrile illness presenting to urban and rural community-based health posts had high levels of anti-leptospiral antibodies suggestive of acute leptospirosis. This finding was consistent with the observation that exposure to *Leptospira* is common in daily life in the tropical setting [10]. We hypothesized that concentrations and species of pathogenic *Leptospira* in environmental surface waters would be associated with both the risk of acquiring leptospirosis and the risk for severe disease.

In the past, a major impediment to assessing environmental risk for leptospirosis exposure has been the difficulty of isolating pathogenic *Leptospira* from surface waters, attributable at least in part to the observation that non-pathogenic (saprophytic) leptospires outgrow pathogens in culture. Other methods of identifying *Leptospira* in environmental water and soil sources, including culture and direct animal inoculation, are time-consuming and laborious, and run the risk of missing the bacteria altogether [21,22]. To overcome these limitations*,* we used a quantitative real-time PCR assay to determine the presence of pathogen-related *Leptospira* in environmental water samples from rural and urban sites in the Peruvian Amazon. We then used molecular taxonomical approaches to investigate the link between the identity of environmental leptospiral sequences and human leptospiral isolates obtained during a prospective, population-based study of acute febrile illness.

## Methods

### Description of Study Area

The city of Iquitos is a major tourist destination as well as an important local market town and small industrial center. It is approximately 120 meters above sea level in the Amazon River basin near the juncture of the Ucayali and Napo Rivers (73 °W, 3 °S) in the department of Loreto, in northeastern Peru (http://www.wikimapia.org/#y=-3765768&x=-73265676&z=14&l=0&m=a). It has a population of approximately 400,000 and a surrounding rural population of 474,000. The climate is tropical: rainfall averages 3 m/y and temperatures range from 21.8 °C to 31.6 °C; the city is surrounded by a vast expanse of humid tropical rainforest.

**Figure 1:**
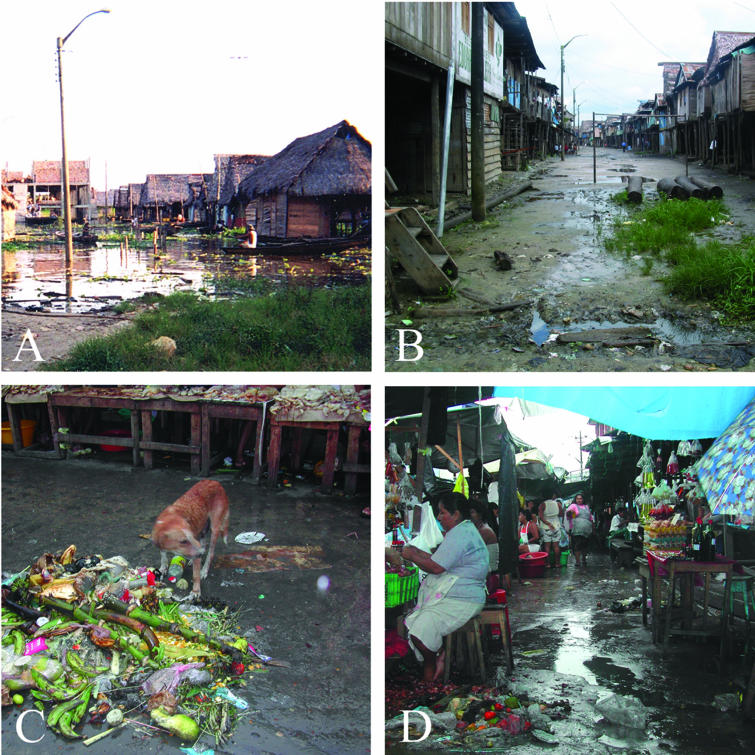
The Urban Slum Environment of the Belen District of Iquitos (A and B) Typical houses in the Belen district, built on stilts to avoid flood waters when the river rises. (C) Typical “sanitation” in the Belen Market area that attracts scavenger animals such as dogs and rats. (D) A typical view of the Belen Market area where commerce is conducted. Rats are often seen beneath tables and in gutters. Rats are so abundant in the market areas that they are commonly seen during the day although they are primarily nocturnal. (With kind permission of Springer Science and Business Media)

Belen (Figure 1), with a population of approximately 40,000, is an urban slum area located on the shoreline of the Itaya River; it floods annually during the rainy season (December through May). Most of its houses are built of wood over stilts or on floats, and are surrounded by open sewers, gutters, rainwater collections, and river-water puddles. Belen's water supply comes from Iquitos, but few households have access to piped water. Most inhabitants buy water from the households that have access to that supply or use river water for their daily needs. There is no closed sewage system. Open sewers and gutters run along the housing area, draining into the river. The market of Belen occupies approximately 25% of the district's area; its sanitation conditions are very poor; open garbage piles, sewers, and rainwater collection puddles are common (Figure 1). Rats, stray cats, and dogs are seen on a daily basis in the market.

**Figure 2:**
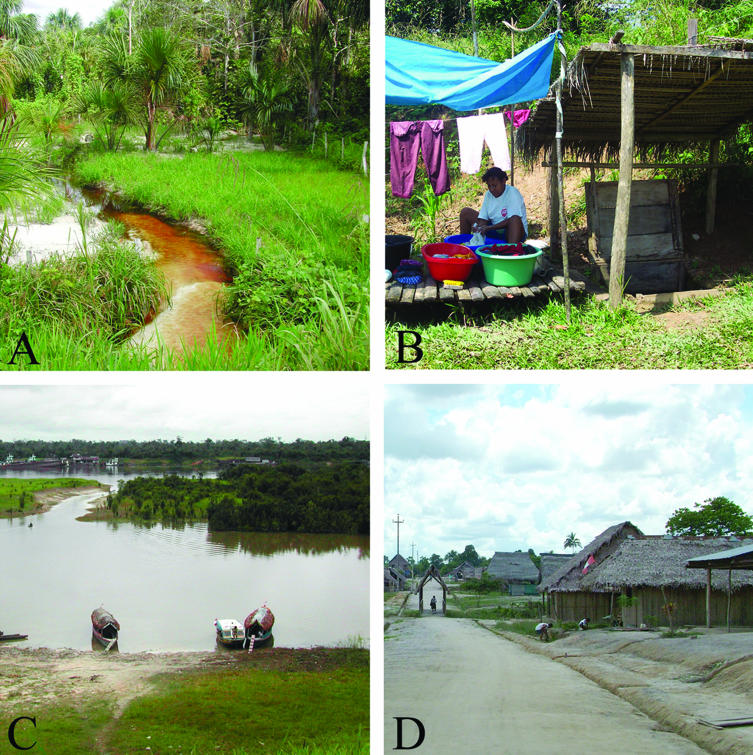
The Rural Village of Padrecocha, Outside of Iquitos (A) A quebrada (stream), strongly associated with leptospirosis transmission. (B) A typical well in close association with activities of daily living. (C) The pond of Padrecocha (the cocha) adjacent to the river. (D) Flooding in the village during the high river season after heavy rains. (With kind permission of Springer Science and Business Media)

Padrecocha (Figure 2) is a rural community near Iquitos, located north of the city along the Nanay River, a tributary that branches from the Amazon River 15 km downstream from Iquitos. The population of this village is approximately 1,500. Most inhabitants live in brick houses, and their water supply comes from wells and local streams. There is no sewage system; most households have pit latrines. Livestock (mostly pigs, chickens, and cattle) run freely through the village and its streams, along with stray dogs and cats; the inhabitants observe rats frequently.

### Description of Study Sites

Water samples were collected from both locations from February to April 2004. In Belen, samples were collected from two areas, the Belen Market area and the housing area (Belen Living area). From the market area, 78 water samples were collected from puddles and gutters. From the living area, 114 water samples from puddles, gutters, pooled rainwater, and river shoreline water were collected; these samples were all collected in the vicinity of the shoreline of the slow-moving river, where mud, gutters, and water collections were available. In rural Padrecocha, a timed water collection approach was used to determine the persistence of leptospiral contamination of the water sources. Water sources (24 total) were selected according to spatial distribution and sampled sequentially over ten different time points; these sources included fresh water wells (17), a fish farm (one), and slow-moving fresh water streams (six), totaling 236 samples from that village.

### Prospective Hospital-Based and Population-Based Study of Human Leptospirosis in the Iquitos Region

In the context of a prospective study of leptospirosis patients conducted at the Hospital de Apoyo and rural outposts (Moralillo, Varillal, and Zungarococha villages) in and around Iquitos from 01 June 2003 to 01 March 2004, 21 leptospiral strains from 600 febrile patients (3%) screened were isolated during the study period; the overall rate of diagnosis of acute leptospirosis by serological criteria in these sites was approximately 50% and did not differ between urban and rural sites [9]. The clinical study protocol has been described [9].

### Molecular Analysis of Leptospiral Sequences from Environmental Water Samples

Water samples were tested for the presence of leptospiral DNA by a quantitative real-time PCR assay confirmed by nested 16S rDNA PCR and by sequencing those products.

#### Water collection and DNA extraction.

Samples of water (50 ml) from each site were collected in sterile polypropylene centrifuge tubes. The samples were centrifuged at 3,000 *g* for 30 min at room temperature. DNA from the pellet was extracted using the QIAamp DNA Mini Kit (Qiagen, Valencia, California, United States) following the manufacturer's protocol for urine DNA extraction.

#### Real-time PCR.

A published TaqMan assay targeting the 16S ribosomal leptospiral gene for pathogen-related *Leptospira* [23] was performed on-site in our Iquitos laboratory using an Opticon2 real-time PCR machine (MJ Research, BioRad, Hercules, California, United States); the assay protocol was modified from the published version by using the fluorescent probe at a final concentration of 0.2 μM, primers at a final concentration of 0.5 μM, and a 20 μl reaction volume. Standard curves for quantification were made using Leptospira interrogans serovar icterohemorrhagiae strain M20. Standards were prepared as follows. Leptospires were counted using a Petroff-Hauser (Hauser Scientific, Hosham, Pennsylvania, United States) counting chamber and serially diluted with sterile double-distilled H_2_O to make 10^8^ to 10^0^ leptospires/ml. Genomic DNA was subsequently prepared using the DNeasy Tissue Kit (Qiagen). Standards were run in triplicate to generate the standard curve. A negative result was assigned where no amplification occurred before 40 cycles. Controls lacking template (water only added to reaction mix) were included to detect the presence of contaminating DNA. In addition, those water samples amplifying after the last reliable standard but prior to a threshold cycle (Ct) of 40 cycles on the TaqMan real-time PCR assay were considered suspicious for pathogen-related *Leptospira* and reamplified with the following nested-PCR primers: lepto16S11f (5′-GGCGGCGCGTCTTAAACATGC-3′) and lepto16S1338r (5′-TGTGTACAAGGTCCGGGAAC-3′). These primers were designed for this study using 16S rDNA sequences (*n* = 39; Table 1) retrieved from GenBank aligned using CLUSTALW 1.83 (http://www.ebi.ac.uk/clustalw). Primers were selected from conserved regions of the 16S rDNA genes of pathogen-related and intermediate leptospiral species at the 5′ and 3′ ends of the sequences. Briefly, 5 μl of DNA was added to the 45 μl HotStarTaq Master Mix (Qiagen), providing final concentrations of 0.2 μM of each primer. “No template” controls were also included to detect the presence of contaminating DNA. Amplification was conducted in a DNA Engine PCT-200 Peltier Thermal Cycler (MJ Research). The amplification protocol consisted of 95 °C for 16 min, followed by 35 cycles of amplification, each cycle consisting of 94 °C for 30 s, 56 °C for 1 min, and 72 °C for 2 min.

**Table 1:**
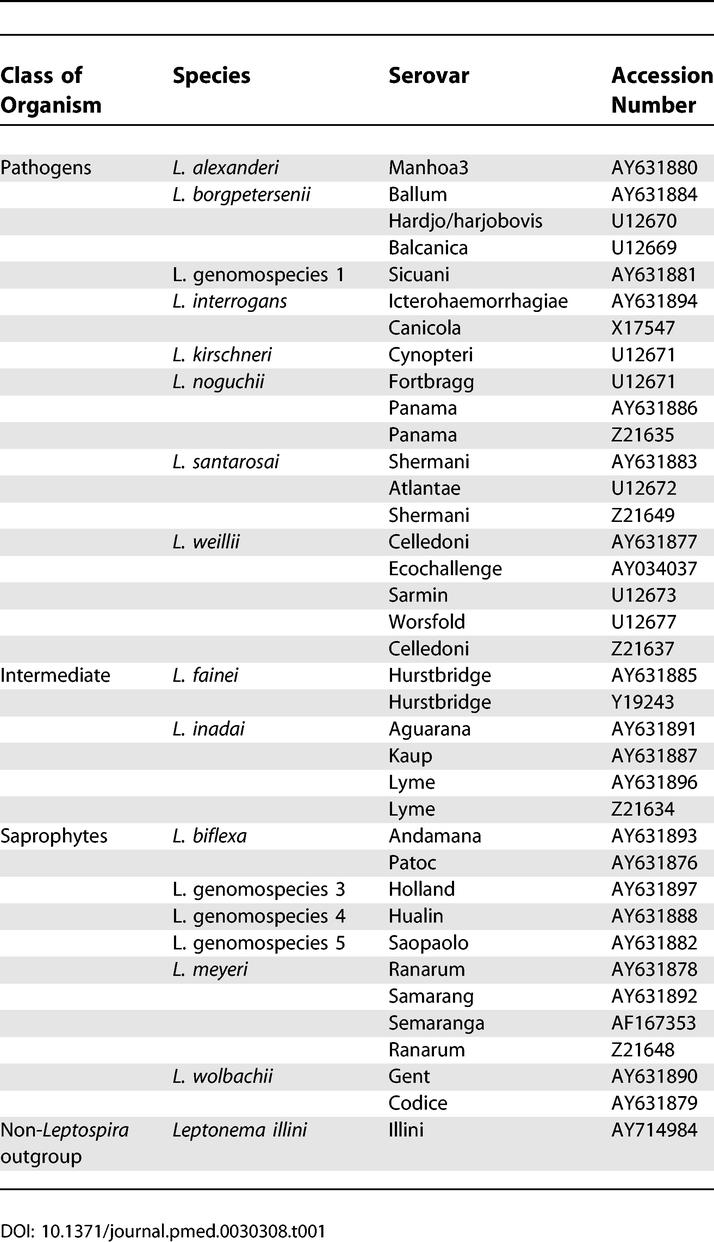
GenBank Accession Numbers of Leptospiral 16s rRNA Gene Sequences Used in this Study

#### Leptospiral 16S ribosomal RNA gene PCR amplification and sequencing.

Total genomic DNA from 35 randomly selected water samples was amplified and cycle-sequenced. Template (5 μl) was amplified using the primers fD1/rD1 as described previously [13]. PCR products were purified from 1.0% agarose gels in TAE buffer using the QIAEX II gel extraction kit (Qiagen) according to manufacturer's directions, diluted 1:100 in sterile double-distilled H_2_O, subjected to a second round of amplification using the nested primers lepto16S11f and lepto16S1338r with the protocol described above, and then cloned into the pCR2.1-TOPO vector (Invitrogen, Carlsbad, California, United States). Recombinant plasmids bearing the nested PCR products were then transformed into TOP10 cells (Invitrogen) and plated on LB agar containing 100 μg/ml ampicillin. Individual clones were then hand-picked and grown overnight in LB broth containing 100 μg/ml ampicillin. Plasmid DNA from these clones was purified using the QIAprep Spin Miniprep Kit (Qiagen) and cycle sequenced. Sequencing was performed on an ABI 3100 automated sequencer (PerkinElmer, Wellesley, California, United States) using the following forward primers: lepto16S11f, lepto16S505f (5′-TCATTGGGCGTAAAGGGTG-3′), and lepto16S1006f (5′-TCAGCTCGTGTCGTGAGATG-3′) and the reverse primer lepto16S1338r. Reaction conditions were according to the manufacturer's directions.

### Molecular Analysis of Leptospiral Sequences from Human Isolates

Leptospira cultures from 21 human isolates were DNA-extracted, and the bacterial 16S rDNA fragments were PCR-amplified, inserted into plasmid vectors, amplified in *E. coli,* and cycle sequenced using the same procedures described above.

### Phylogenetic Analysis of 16S rRNA Gene Sequences from Water Samples and Human Isolates

Sequences were assembled using the program CAP3 (http://pbil.univ-lyon1.fr/cap3.php) then aligned using CLUSTALW v1.83 with default parameters. *Leptospira* 16S rRNA sequences from GenBank were used as controls in the tree (Table 1). Sequences derived from isolates and water samples were analyzed simultaneously and with identical parameters for the nucleotide substitution model. Missing (gaps) and ambiguous characters were excluded from the analysis. However, a separate data partition was included whereby gaps were coded as binary state data; with gap characters were coded as 1 while all others were coded as 0. This data partition was analyzed using the restriction site model as implemented in MrBayes (specific application to leptospiral taxonomy [24]). A phylogenetic dendrogram was generated using MrBayes v3.1.2 running for 3,000,000 generations. The data were analyzed using the GTR nucleotide substitution model with gamma-distributed rates and proportion of invariant sites. The resulting datasets were then analyzed using flat priors for the substitution rate parameters.

### Statistical Analysis

Statistical analysis was done using the statistical software package GraphPad Prism 4 for Macintosh (GraphPad Software, San Diego, California, United States). Sample positivity between locations and sources were analyzed by chi-square. The difference in the average of the bacterial counts was analyzed by ANOVA using Newman-Keuls multiple comparison test and with the unpaired t-test using Welch correction. The timed sampling analysis used the chi-square test. The Fisher's exact test was used to analyze the association of pathogenicity (pathogen versus intermediate versus saprophyte) and location.

## Results

### Location and Frequency of PCR Detection of *Leptospira* in Surface Water Sources

In Belen, 192 water samples were collected to test for the presence of pathogen-related leptospiral DNA. From the market area of Belen, 53 (67.9%) of 78 water samples were positive for leptospiral DNA. From the living area, 38 (33.3%) samples of 114 were also positive. The difference in the rate of positivity between the living and markets areas was statistically significant (*p* < 0.0001). In Padrecocha, 60 (25.4%) of 236 samples were PCR-positive. The rate of positivity differed among the Belen market area, the Belen living area, and the Padrecocha sites (χ^2^ = 46.69, *p* < 0.001).

### Association of Type of Water Source and the Presence of *Leptospira*

In the market area of Belen, all positive samples came from open gutters and puddles. In the living area of Belen no difference was observed between the source of the sample and the rate of positivity (χ^2^ = 1.62, *p* > 0.05). In Padrecocha, of 60 positive samples, 34 (57%) were collected from streams (six collection points on two streams) and 26 (43%) were collected from underground sources (17 wells); significantly more stream samples than well samples were positive (χ^2^ = 37.99, *p* < 0.001).

### Quantification of *Leptospira* in Environmental Water Samples

**Figure 3:**
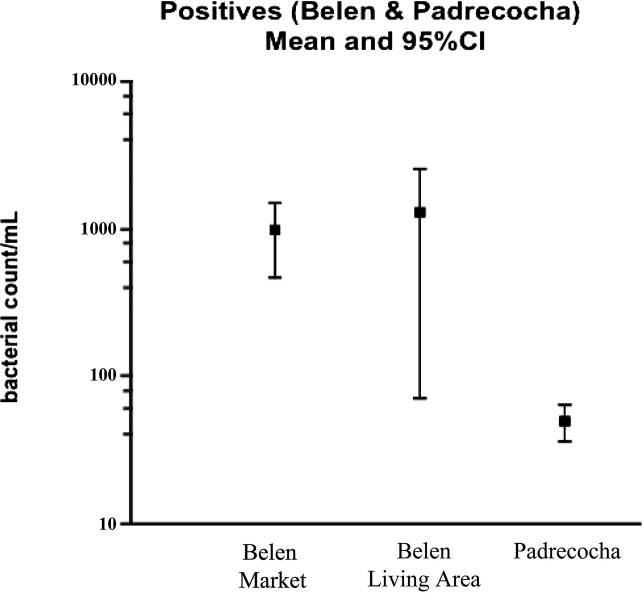
Quantification of *Leptospira* in Belen Versus Padrecocha Bars indicate 95% confidence intervals. Analysis of variance, *F* = 8.406, *p* = 0.0003. Newman-Keuls multiple comparison test: Padrecocha versus Belen market, *p* < 0.001; Padrecocha versus Belen living area, *p* < 0.01; Belen market area versus Belen living area, *p* > 0.05.

**Figure 4:**
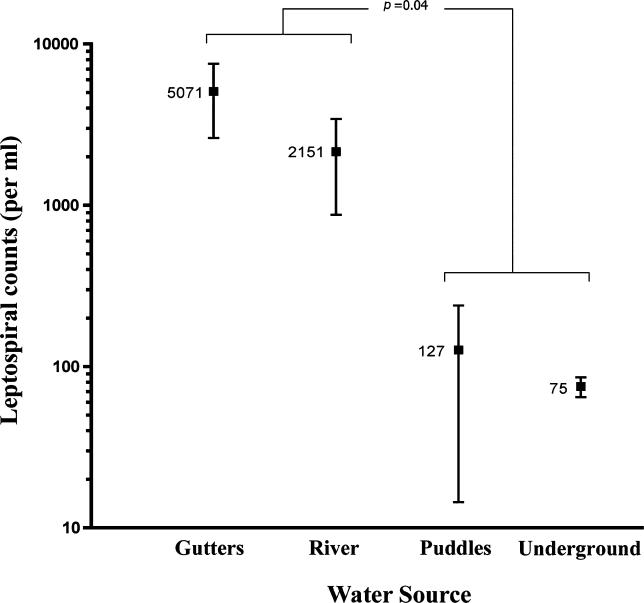
Comparison of Leptospiral Counts in the Belen Living Area, According to Water Source Means and 95% CIs are shown. Open gutters and river samples showed significantly higher bacterial counts than the samples from puddles and underground sources (unpaired t-test with Welch's correction: *t* = 2.284, *p* < 0.05).

In the market area of Belen, the bacterial count range for the 53 positive samples was 2–8,032 leptospires/ml (mean, 954 leptospires/ml [95% confidence interval (CI), 463–1,445 leptospires/ml]). In the living area of Belen, the counts for the 38 positive samples were 2–17,147 leptospires/ml (1,286 leptospires/ml [110–2,461 leptospires/ml]). In Padrecocha, the counts for the 60 positive samples were 1–228 leptospires/ml (49 leptospires/ml [35–62 leptospires/ml]). The ANOVA test showed a statistically significant difference between the bacterial counts of the three locations (*F* = 8.406, *p* < 0.001). In the Newman-Keuls multiple comparison test, no significant difference in the bacterial counts between the living and market areas of Belen was observed (*p* > 0.05), but a significant difference was found between the bacterial counts of these two areas and Padrecocha (*p* < 0.01) (Figure 3). In a subset of 15 positive samples of the living area of Belen, the samples from open gutters and the river showed significantly higher leptospiral counts than the samples from puddles and underground sources (unpaired t-test with Welch's correction; *t* = 2.284, *p* < 0.05) (Figure 4). In Padrecocha, no statistically significant difference existed between the bacterial counts of the positive samples from wells and streams (unpaired t-test; *t* = 1.154, *p* > 0.05).

### Presence of *Leptospira* in Sequential Samples from the Rural Village of Padrecocha

**Figure 5:**
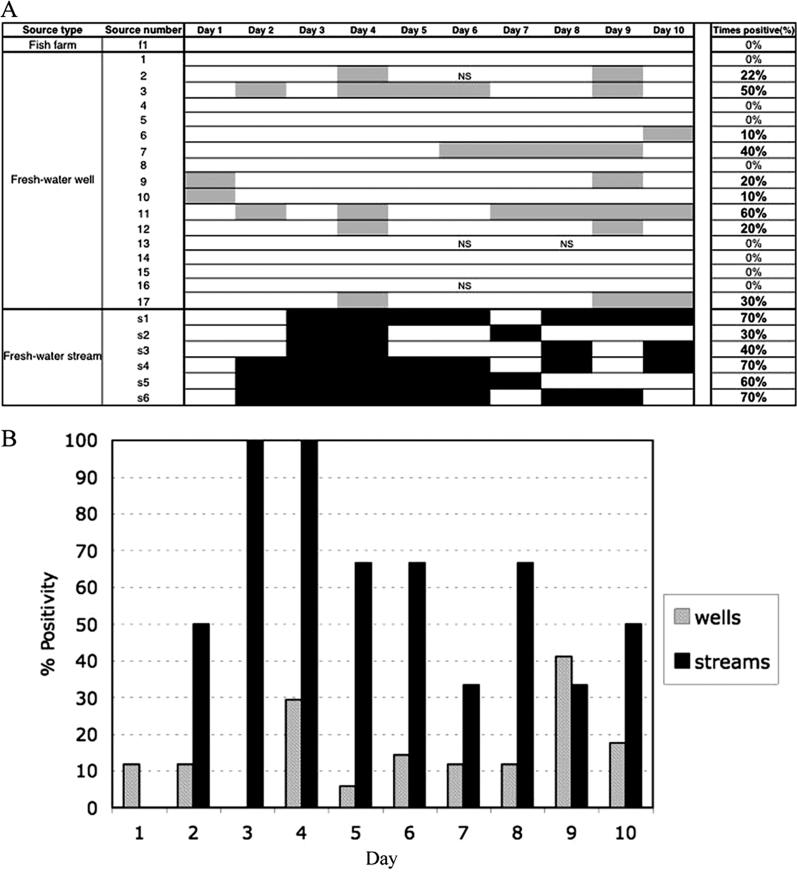
Padrecocha Sequential Sampling A total of 17 wells and six stream sampling points were tested over time. Filled boxes indicate PCR-positivity on that day (A). Bars indicate percentage of positivity of wells and stream sampled points for each tested day (B). The streams had a statistically significant higher frequency of positivity over time than the wells (χ^2^ = 133.74, *p* < 0.001). NS, not sampled.

All sampling points in Padrecocha streams were PCR-positive at least once during the sequential sampling. In contrast, of 17 wells sampled in Padrecocha, only nine (52.9%) were PCR-positive at least once during repeated sampling. None of the samples from the fish farm were positive. The average number of positive time points for the streams was 5.7 times (57% of the sampling time points, range three to seven times), and the average number of positive time points for the wells was 2.9 times (29% of the sampling time points, range two to six times). The streams were more often positive over time than the wells (χ^2^ = 133.74, *p* < 0.001) (Figure 5).

### Molecular Identification of Leptospiral Species by DNA Sequencing of Nested 16S PCR Products

**Figure 6:**
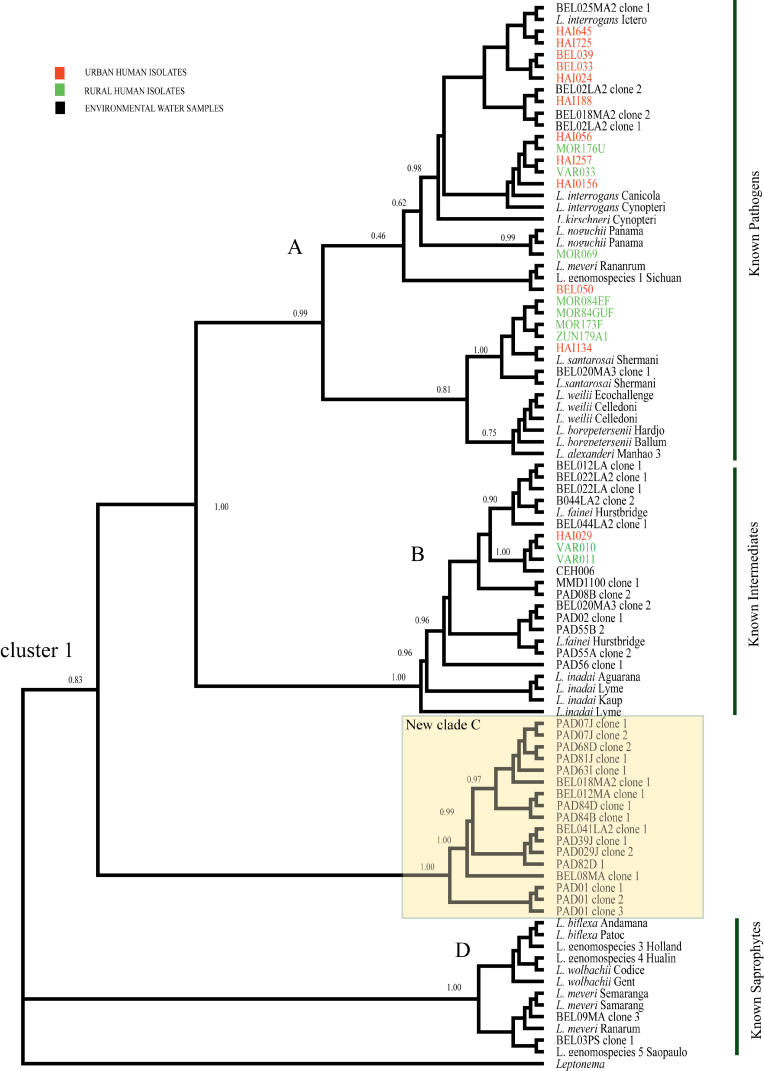
Phylogenetic Analysis of Leptospiral 16s rRNA Gene Sequences Sequences represent human isolates and clones from environmental water samples obtained during a prospective study of leptospirosis in Peru using a Bayesian approach. The top cluster (A) comprises strains of the most pathogenic leptospiral species; also included are intermediates (B), the new clade (Clade C) (C), and saprophytes (D). Numbers at each node represent the posterior probability of the given node. Nodes without support values had less than 50% posterior probability, but are consistent with the overall branching of the tree. BEL indicates isolates obtained from patients seen in Belen, a busy riverside market community frequented by Rattus spp.; HAI indicates isolates obtained from patients seen at the Hospital de Apoyo; both are within the urban/periurban center of Iquitos (highlighted in red). Human isolates from rural areas (green) have been designated MOR (Moralillo), VAR (Varillal), and ZUN (Zungarococha) consistent with the location of the villages at which the patients were seen and the cultures obtained. Clones from water samples from Belen have been designated BEL (LA, living area; MA, market area), and samples from Padrecocha as PAD. Nodes highlighted with an asterisk (*) have support values less than 50%, but are consistent with the overall branching of the tree. Padrecocha is a rural area with a significantly lower rat population density but with many pigs roaming free.

Of 21 consecutive isolates (Figure 6) from leptospirosis patients from urban and periurban Iquitos (12 cases), and outlying rural areas (nine cases), L. interrogans isolates were detected almost exclusively in urban/periurban habitats (9 [82%] of 11 isolates). Leptospiral isolates from rural areas were primarily other leptospiral species (7 [70%] of 10), particularly L. santarosai and L. noguchii. Most cases were caused by L. interrogans (11 [52%] of 21) and L. santarosai (5 [24%] of 21). The relatively high proportion of L. interrogans serovars was associated with urban acquisition of the spirochete (Fisher's exact test; *p* = 0.03) (Table 2).

**Table 2:**
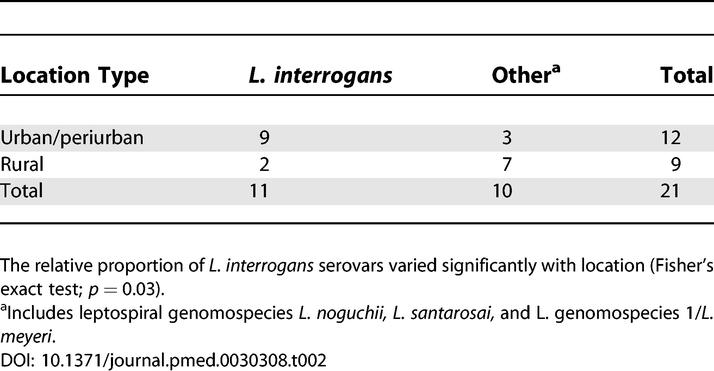
Distribution of L. interrogans Strains with Location

In addition to the 21 human isolates, 35 clones of 16S ribosomal RNA gene sequences were obtained from PCR-positive surface water samples that were randomly selected for sequencing (Figure 6). Of these clones, 16 were from the urban slum of Belen and 19 from the rural village of Padrecocha. Bayesian phylogenetic analysis demonstrated that these 16S gene leptospiral sequences could be divided into four major monophyletic groups: known pathogens, *Leptospira* considered to be intermediately pathogenic (hereafter termed “intermediates”), known saprophytes, and a previously unreported group (termed “clade C”) composed only of clones from this study (Figure 6). There was strong support (95%) for the inclusion of this new monophyletic group within the subgroup of pathogen-related/intermediate leptospiral strains. Most nodes were well supported with all but three clades receiving strong support (>75% posterior probability).

Analysis of most (34 [97.1%] of 35) leptospiral DNA sequences from the water samples indicated that they arose from pathogen-related/intermediate leptospires (Figure 6). Of the Belen clones five clustered with the pathogens, four were identical to published sequences from *L. interrogans/L. kirschneri,* and the other was most similar to L. santarosai. The remaining clones from Belen clustered with the intermediate strains (six sequences), clade C (four sequences), and saprophytic strains (one sequence) (Figure 6).

All sequenced 16S clones from Padrecocha clustered with the intermediate strains L. fainei and L. inadai (six sequences) or a previously undescribed clade (clade C, 13 sequences). The intermediate cluster also included two strains that had been previously detected in Peru (isolates CEH006 and MMD1100), and an uncultured leptospire detected previously in a patient dying from severe leptospirosis (HAI332) [10]. CEH006 was isolated from Rattus norvegicus in Belen while clone MMD1100 was only detected by PCR in the kidney of a bat *(Uroderma bilobatum)* collected near the village of Varillal 15 km from Iquitos [24]. Consistent with these findings, we have recently obtained 16S rRNA gene sequencing data from more than 20 isolates obtained from cattle and pigs from the Iquitos region. These data indicate that L. santarosai is the predominant infecting species of these livestock, while L. interrogans predominantly is found in peridomestic *Rattus* rat species (MAM, CBC, CAG, and JMV, unpublished data).

Clones that clustered within the pathogen-related group were significantly more likely to be recovered from Belen than in rural sites (*p* = 0.013, Fisher's exact test) (Table 3). Three 16S rRNA gene sequences from water samples (BEL25MA2, BEL18MA2, and BEL02LA2) were identical to those of human isolates (HAI024, HAI188, HAI645, and HAI725). These human isolates were typed as L. interrogans serovar Icterohaemorrhagiae*.*

**Table 3:**
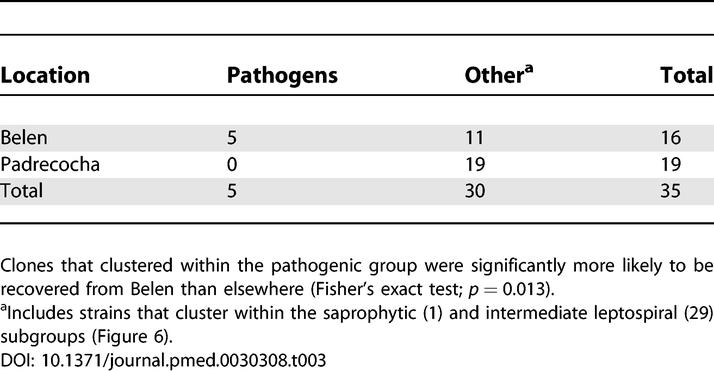
Distribution of Pathogenic Versus Saprophytic/Intermediate Leptospiral Strains by Location

## Discussion

We describe a new approach to identifying and quantifying pathogen-related leptospires in environmental water sources. A quantitative molecular and taxonomical method based on combining real-time reverse transcriptase PCR and DNA sequencing provided an important measure of human risk both for acquiring leptospirosis and for developing severe disease. Urban slums in Iquitos, Peru had high concentrations of pathogenic *Leptospira* and *L. interrogans-*type leptospires in surface waters such as those found in the market area and along the riverfront. In contrast, a rural area near Iquitos had lower concentrations of pathogenic *Leptospira* in surface waters such as wells. These *Leptospira* were less likely than those in the urban environment to be L. interrogans (the more highly pathogenic species). The concentration and species of leptospires in the water sources correlated with risk of severe human leptospirosis.

The outcome of human infection by *Leptospira* can be divided into host and microbiological factors. Different genetic backgrounds, for example, containing mutations altering the major histocompatibility complex [25] or innate immunity [26] might modulate outcome of human infection. Virulence factors of *Leptospira* may differ among infecting strains, and which ones may be variably expressed in different mammalian hosts [27] are important factors likely to modulate outcome of infection. Here we show that the type and quantity of leptospires in surface waters that are potential sources of human infection are also related to the outcome of infection. This approach to the identification of uncultivated pathogens in environmental surface waters may be applicable to assessing risk for other water-borne infectious diseases such as salmonellosis, shigellosis, tularemia, and cryptosporidiosis.

We used molecular taxonomical approaches to directly link both the concentration and the species of pathogen-related *Leptospira* in environmental surface waters to the types of *Leptospira* that infect humans. We found that L. interrogans species, more commonly associated with severe human leptospirosis, were found significantly more often in urban than in rural environmental water sources where species other than *L. interrogans,* such as L. santarosai (associated with pigs and cattle) and intermediately pathogenic Leptospira spp. (of uncertain mammalian reservoirs) predominated. This differential distribution of leptospiral sequences in environmental water sources mirrored that of human isolates from urban and rural settings. We interpret these data to indicate that one important factor determining the risk of severe human leptospirosis, together with pathogen-host immune interactions, is both leptospiral species and concentration of pathogen-related *Leptospira* present in environmental surface waters. These findings are important because these factors are targets amenable to public health intervention, more so than bacterial or host factors. After validation in other settings and on a larger scale, the molecular identification and quantification of *Leptospira* in environmental water samples may be useful for guiding environmental remediation to prevent leptospirosis in endemic regions.

Epidemiological approaches to identifying risk factors for acquiring leptospirosis have traditionally focused on assessing potential zoonotic reservoirs, behavioral risk factors, and various sociodemographic features of human populations. While other investigators in the past have sought to identify pathogen-related leptospires in environmental sources using culture techniques, this approach has proven to be difficult and unreliable, being hindered in two ways: (1) saprophytic leptospires predominate in the environment that are morphologically similar to pathogen-related *Leptospira* but grow faster; and (2) the culture methods for identifying pathogen-related *Leptospira* are laborious, time-consuming, and insensitive. However, while DNA amplification techniques have been published and used for diagnosis of leptospirosis, they cannot identify all the leptospiral serovars in one reaction nor distinguish leptospiral species.

In both urban and rural settings, anthropogenic influences on ecology and environment seem to drive leptospiral transmission to humans. The quantification of leptospiral DNA copies in the water samples tested shows that in the living area of Belen, gutter and stagnant river water samples had higher concentrations of leptospires than did rainwater collection and underground water sources. This finding can be explained by the presence of chronically infected rodents, dogs, and pigs contaminating these water sources, as opposed to the underground sources, which are often protected from animal contact. In rural Padrecocha, the freshwater stream samples were positive most of the time compared to the freshwater well samples. This may be explained by the fact that many inhabitants use chlorine for water decontamination and detergent and soap for clothes washing and bathing near the wells; leptospires are inhibited at low detergent concentrations [11]. Alternatively, it is possible that the *Leptospira-*negative wells were relatively protected from environmental runoff, thus avoiding higher level contamination from animal urine. The high and persistent positivity of the freshwater streams (especially in the stream that runs through the village) may be due to the presence of cattle and pig farms and also of wild mammals along the streams. These findings are also important because they can explain the persistent sources of transmission in these populations; activities of daily living are strongly linked to water exposure (fishing, bathing, clothes washing, etc.).

In a previous cross-sectional study, the prevalence of anti-leptospiral antibodies was found to be significantly higher in residents of Belen (28%) than in rural areas near Iquitos (17%) [10]. This observation suggests the possibility that protective immunity against severe disease from repeated infection may develop in areas with high leptospirosis transmission, especially if high frequency of infection leads to cross-serovar protection. These findings are also consistent with the finding that most of the cases of severe leptospirosis seen in Iquitos come from urban districts of the city rather than from periurban slums such as Belen or rural areas such as Padrecocha [9]. Such possibilities need to be tested in prospective, population-based studies of acquired immunity to symptomatic leptospirosis.

This study had several limitations. First, because of technical limitations in leptospiral cultivation, we were not able to identify leptospiral serovars in the water samples. Second, the real-time PCR assay used in this study, previously reported to be specific for the detection of pathogen-related *Leptospira* [23], is not as specific as previously reported—we were able to identify noncultivated leptospires of unknown pathogenicity. In fact, we were able to identify new *Leptospira* of unknown pathogenicity, since none has yet been identified in mammals. We have provisionally termed this group of *Leptospira* “clade C.” Because these clade C sequences were mostly found in rural settings, this quantitative real-time PCR cross-reactivity problem does not invalidate the basic finding of higher densities of pathogens in ground water from urban settings. Our data do confirm published reports, in that all positive samples we identified were confirmed by sequencing, even those samples that had high Ct values beyond the limit of detection in the standard curve (low leptospiral counts considered as suspicious [unpublished data]). Other problems with this methodology are expense and availability of the equipment. However, the advantages of this method make possible the study of large areas in a short period of time, such as we studied here, allowing for the rapid deployment of environmental risk assessment in endemic regions, particularly in the setting of epidemics. Finally, we did not use high-throughput sequencing of 16S rRNA gene fragments to provide a large-scale representation of leptospiral sequences in the water samples. Such a study is ongoing.

Identifying the strains present in environmental water samples may provide insight into the relevance of local mammal species to the dissemination of leptospires and occurrence of human leptospirosis in Iquitos. For example, most strains sequenced from Padrecocha belonged to possibly new intermediate leptospiral species (MAM, JN Ricaldi, and JMV, unpublished data). 16S rRNA gene sequences found in water sources have also been detected in cattle, pigs, and bats, suggesting that in this rural setting, these animals may contribute to human leptospirosis (CBC, MAM, and JMV, unpublished data). In Belen, where rat populations are dense, we detected 16S rDNA gene sequences similar to several different L. interrogans serovars. Rats common in Belen *(R. norvegicus* and *R. rattus)* carry L. interrogans with identical 16S rDNA gene sequences (unpublished data), suggesting the potential importance of these animals to the transmission of leptospirosis in the area. In addition, of 21 cases of leptospirosis recorded among patients from urban and periurban Iquitos and outlying rural areas, we noted that L. interrogans clones were detected almost exclusively in urban/periurban habitats, while clones from rural areas significantly comprised other leptospiral genomospecies, including L. santarosai and *L. noguchii,* which have been shown to be carried by cattle and wildlife reservoirs (CBC, MAM, and JMV, unpublished data). Furthermore, in this study, L. interrogans was found to be the primary cause of severe leptospirosis in Iquitos [9]. Taken together, these observations suggest that real-time detection and sequencing are potentially useful for determining the significance of local reservoirs of leptospires, and that an association with rats is a significant risk factor for acquiring severe leptospirosis in Iquitos.

Leptospirosis continues to be overlooked globally as a major public health threat. Not only does leptospirosis have highly variable, nonspecific clinical presentations, but diagnosis is difficult and requires a high index of suspicion particularly with regard to potential environmental exposure. In the Amazon region of Peru, leptospirosis is an important cause of morbidity and mortality. More effective, timely, and efficient methods need to be deployed for rapid clinical diagnosis and for environmental risk assessment. PCR-based detection and identification of leptospiral DNA, which avoid the problems inherent to isolation of fastidious organisms from contaminated sources, can be useful for assessing and monitoring risk of human populations to *Leptospira*-contaminated water. The methodologies presented here need to be validated further in prospective studies in different regions of the world where leptospirosis is common. The researchers conducting these studies should provide their data to Ministries of Health and policy-making agencies critical for justifying public health and occupational medicine campaigns to control leptospirosis transmission. General health promotion should also continue to use common-sense strategies such as wearing shoes and eliminating trash from the home and local environments.

## Supporting Information

Alternative Language Abstract S1 Translation of Abstract into Spanish by Christian Ganoza (25 KB DOC)

Protocol S1 Appendix (21 KB DOC)

### Accession Numbers

The new leptospiral sequences described in this paper have been deposited in GenBank (http://www.ncbi.nlm.nih.gov) under the following accession numbers: BEL012LA_1_(DQ522176); BEL012MA_1 (DQ522177); BEL018MA2_1 (DQ522178); BEL018MA2_2 (DQ522179); BEL020MA3_1 (DQ522180); BEL020MA3_2 (DQ522181); BEL022LA2_1 (DQ522182); BEL022LA_1 (DQ522183); BEL025MA2_1 (DQ522184); BEL02LA2_1 (DQ522185); BEL02LA2_2 (DQ522186); BEL03PS_1 (DQ522187); BEL044LA2_1 (DQ522188); BEL044LA2_2 (DQ522189); BEL08MA_1 (DQ522190); BEL09MA_3 (DQ522191); BEL041LA2 (DQ522192); BEL050 (DQ522193); CEH006_1 (DQ522194); HAI156 (DQ522195); HAI024 (DQ522196); HAI029 (DQ522197); HAI056 (DQ522198); HAI134 (DQ522199); HAI188 (DQ522200); HAI257 (DQ522201); HAI645 (DQ522202); HAI725 (DQ522203); MOR069 (DQ522204); MOR084EF (DQ522205); MOR173F (DQ522206); MOR176U (DQ522207); MOR84GUF (DQ522208); PAD01_1 (DQ522209); PAD01_2 (DQ522210); PAD01_3 (DQ522211); PAD029J_2 (DQ522212); PAD02_1 (DQ522213); PAD07J_1 (DQ522214); PAD07J_2 (DQ522215); PAD08B_2 (DQ522216); PAD39J_1 (DQ522217); PAD55A_2 (DQ522218); PAD55B_2 (DQ522219); PAD56_1 (DQ522220); PAD63I_1 (DQ522221); PAD68D_2 (DQ522222); PAD81J_1 (DQ522223); PAD82D_1 (DQ522224); PAD84B_1 (DQ522225); PAD84D_1 (DQ522226); VAR010 (DQ522227); VAR011 (DQ522228); VAR033 (DQ522229); ZUN179A1 (DQ522230); BEL033 (DQ522231); BEL039 (DQ522232).

## **Abbreviations**v

CI: confidence interval
