# Supplementary material for: Determining Risk for Severe Leptospirosis by Molecular Analysis of Environmental Surface Waters for Pathogenic Leptospira
Source: PLoS Med. 2006 Aug 22;3(8):e308. doi: 10.1371/journal.pmed.0030308 (PMC1551915; doi:10.1371/journal.pmed.0030308)
Supplement: Supplementary file 1 [file pmed.0030308.sd001.doc]

**Resumen**

En la Amazonía Peruana la leptospirosis severa solo ha sido observada en un contexto urbano, a pesar de que datos previos indican que la incidencia de esta enfermedad es similar en las zonas urbanas como en las rurales. Como potencial explicación para esta observación epidemiológica, probamos la hipótesis de que las concentraciones de leptospiras más virulentas serán mas altas en el agua superficial ambiental de zonas urbanas que en las de zonas rurales. Un ensayo de PCR cuantitativo en tiempo real fue usado para comparar los niveles de leptospiras en agua superficial ambiental entre zonas urbanas y rurales en la región de Iquitos de la Amazonía Peruana. Para identificar las especies de leptospiras se utilizo un análisis taxonómico molecular de un segmento de 1200pb del gen de ARN 16S ribosomal de éstas. Las leptospiras patógenas fueron encontradas solamente en fuentes de agua de un barrio pobre urbano (prueba exacta de Fisher, p=0.013). La concentraciones de leptospiras relacionadas a las patógenas fueron mayores en las fuentes de agua urbanas que en las rurales (~10^3^ leptospiras/mL vs. 0.5x10^2^ leptospiras/mL, F=8.406, p<0.05). Secuencias idénticas del gen rARN 16s de *Leptospira interrogans* serovar Icterohaemorrhagiae fueron encontradas en el agua de canales de desagüe del mercado de un barrio pobre urbano y en aislamientos de muestras de humanos, sugiriendo un modo de transmisión especifico desde ratas hacia humanos. En un estudio poblacional prospectivo de pacientes con enfermedad febril aguda, el aislamiento de leptospiras relacionadas a *L. interrogans* en humanos estuvo significativamente asociado a adquisición urbana de la enfermedad (75% de aislamientos urbanos); aislamientos de humanos de otras especies de leptospiras fueron asociados con adquisición rural de la enfermedad (78% de los aislamientos rurales)(análisis de Chi cuadrado, *p*<0.01). La distribución de los aislamientos de leptospiras de muestras de humanos reflejaron la distribución de las secuencias del gen 16s ribosomal de leptospiras de fuentes de agua urbanas y rurales. Estos datos apoyan la hipótesis de que la leptospirosis severa urbana en la Amazonía Peruana esta asociada a mayores concentraciones de leptospiras más patógenas en los lugares de exposición y de transmisión. Esta evaluación combinada de cuantificación y taxonomía molecular en muestras de agua superficial es aplicable a escala global para determinar el riesgo de infección por leptospiras y de enfermedad severa en regiones endémicas.
