# Supplementary material for: Determining Risk for Severe Leptospirosis by Molecular Analysis of Environmental Surface Waters for Pathogenic Leptospira
Source: PLoS Med. 2006 Aug 22;3(8):e308. doi: 10.1371/journal.pmed.0030308 (PMC1551915; doi:10.1371/journal.pmed.0030308)
Supplement: Supplementary file 2 [file pmed.0030308.sd002.doc]

**Supporting Information – Text S1**

**Appendix**

Water sampling strategy

a. Rural setting

All wells and water sources in use by the inhabitants at the time of the study (n=24) were sampled. Most wells were makeshift, not covered, dug in clay-based terrain, between 1 and 1.5 meters wide, and 2-2.5 meters deep. Samples were collected from the surface of the wells as from the surface of stagnant portions of the slow moving streams, near bathing, clothes washing and animal-accessible points.

b. Urban setting

Water was collected from the described sources in a weekly or biweekly schedule, and randomly selected depending on the weather and rain. An average of 30 samples (one from each source) was collected on each study visit.

1. Puddles, gutters and pooled rainwater

These sources were selected for sampling based on their accessibility and on their vicinity to homes (in the living area of Belen) and human activity (in the market). Most of the water sources were located in the vicinity of the main river shoreline, except on rainy days, when pooled rainwater was also collected based on its closeness to homes and playgrounds, and where animals and inhabitants were close. Samples were collected from the surface of these sources; most of them were 2-3 cm deep.

2. River

The sampling points were selected based on their vicinity to homes and to places frequented by locals. Samples were collected from the surface of the stagnant areas of the slow moving river near the floating houses, and from bathing and clothes washing sites as well.
